# Supplementary material for: Splice-Junction-Based Mapping of Alternative Isoforms in the Human Proteome
Source: Cell Rep. Author manuscript; Available in PMC 2020 Jan 15. (PMC6961840; doi:10.1016/j.celrep.2019.11.026)
Supplement: 3 [file NIHMS1546469-supplement-3.zip › DF2/PXD000561/Esophagus-28-Q9Y6M7-NGILASPQSAPGNLDNSKSGEIK.pdf]

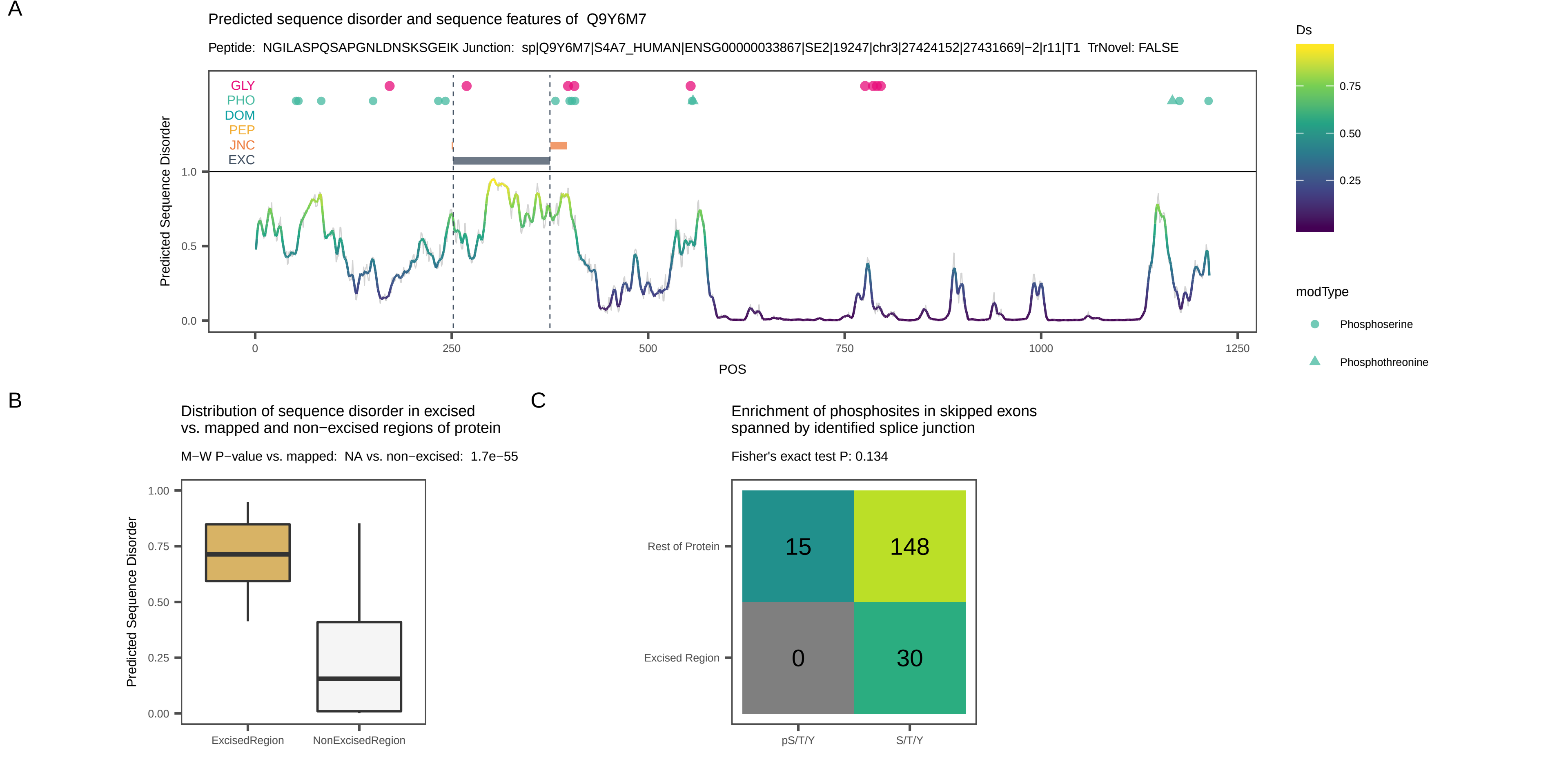
A
Predicted sequence disorder and sequence features of Q9Y6M7
Peptide: NGILASPQSAPGNLDNSKSGEIK Junction: sp|Q9Y6M7|S4A7_HUMAN|ENSG00000033867|SE2|19247|chr3|27424152|27431669|−2|r11|T1 TrNovel: FALSE
GLY
PHO
DOM
PEP
JNC
EXC
Predicted Sequence Disorder
1.0
0.5
0.0
0
250
500
750
1000
1250
POS
Ds
0.75
0.50
0.25
modType
Phosphoserine
Phosphothreonine
B
Distribution of sequence disorder in excised
vs. mapped and non−excised regions of protein
M−W P−value vs. mapped: NA vs. non−excised: 1.7e−55
Predicted Sequence Disorder
1.00
0.75
0.50
0.25
0.00
ExcisedRegion
NonExcisedRegion
C
Enrichment of phosphosites in skipped exons
spanned by identified splice junction
Fisher's exact test P: 0.134
Rest of Protein
Excised Region
15
148
0
30
pS/T/Y
S/T/Y
